# Polynucleotide Injections for Degenerative Meniscal Tears: A Scoping Review of Current Evidence

**DOI:** 10.7759/cureus.105522

**Published:** 2026-03-19

**Authors:** Devansh J Vithlan, Philip Stott

**Affiliations:** 1 Orthopaedics, University Hospitals Sussex NHS Foundation Trust, Brighton, GBR

**Keywords:** degenerative meniscal tear, knee injection, orthobiologics, polydeoxyribonucleotide, polynucleotide, scoping review

## Abstract

Degenerative meniscal tears are a common cause of knee pain and functional limitation, particularly in middle-aged and older adults. Conservative management remains the first-line treatment, with increasing interest in injectable therapies aimed at symptom relief and tissue preservation. Polynucleotide (PN)-based injectables have shown potential benefits in knee osteoarthritis; however, their role in degenerative meniscal pathology remains unclear.

This scoping review aimed to map and synthesise the current clinical evidence on the efficacy and safety of PN injections in adults with degenerative meniscal tears and to identify gaps in the literature.

A scoping review was conducted in accordance with the Preferred Reporting Items for Systematic Reviews and Meta-Analyses extension for Scoping Reviews (PRISMA-ScR) guidelines. Electronic databases, including MEDLINE (Ovid and EBSCO), PubMed, ScienceDirect, SAGE Journals, Wiley Online Library, and the Cochrane Library, were searched from inception to January 2026. Eligible studies were human clinical investigations involving adults with degenerative, non-traumatic meniscal tears treated with PN-based injections and reporting clinical or radiological outcomes. Study selection, data extraction, and risk-of-bias assessment were performed descriptively.

The search identified 72 records, of which 20 full-text articles were assessed for eligibility. One prospective clinical study involving 30 patients met the inclusion criteria. Intra- and perimeniscal PN injections were associated with improvements in pain and functional outcomes, as measured by validated patient-reported outcome scores, including the Visual Analogue Scale, Knee Injury and Osteoarthritis Outcome Score, International Knee Documentation Committee score, and Tegner Activity Scale. No serious treatment-related adverse events were reported. Given the limited number and heterogeneity of eligible studies, quantitative synthesis was not undertaken.

Current evidence on PN injections for degenerative meniscal tears is limited but suggests potential symptomatic benefit and a favourable safety profile. High-quality randomized controlled trials are required to better define the efficacy, comparative effectiveness, and clinical role of this emerging treatment.

## Introduction and background

The knee joint is one of the largest and most anatomically complex joints in the human body. Classified as a synovial hinge joint (a fluid-filled joint that primarily allows bending and straightening movements), it enables fundamental movements such as flexion and extension, while also incorporating rolling, gliding, and rotational components. Despite its structural resilience, the knee remains vulnerable to injury, particularly under torsional or compressive stress.

The joint consists of three primary articulations: the medial and lateral femorotibial joints (between the femur and tibia) and the patellofemoral joint (between the patella and femur), which together facilitate efficient force transmission during locomotion. The menisci are fibrocartilaginous structures interposed between the femoral condyles and tibial plateau and play a crucial role in load distribution [1], shock absorption, joint congruity, lubrication, proprioception, and synovial fluid distribution. The medial meniscus is C-shaped and relatively immobile, while the lateral meniscus is more circular and exhibits greater mobility.

Meniscal anatomy and attachments are fundamental to function and injury susceptibility. The medial meniscus is firmly anchored to surrounding capsuloligamentous structures (the joint capsule and supporting ligaments), including the deep medial collateral ligament and posterior oblique ligament, making it less mobile and more prone to degenerative damage. In contrast, the lateral meniscus covers a larger portion of the tibial plateau and is more mobile due to anatomical features such as the popliteal hiatus (an opening allowing passage of the popliteus tendon) and meniscofemoral ligaments (ligaments connecting the meniscus to the femur).

Meniscal healing potential is limited and closely related to vascularity. The peripheral “red zone” is well vascularized, whereas the central “white zone” is avascular and demonstrates poor intrinsic healing capacity. Blood supply is derived primarily from branches of the popliteal artery via the perimeniscal vascular plexus (a network of small blood vessels surrounding the outer margin of the meniscus), while innervation is similarly concentrated in the peripheral regions. Structurally, the meniscus is composed predominantly of water and type I collagen, with smaller amounts of proteoglycans and a heterogeneous cellular population responsible for extracellular matrix maintenance. Despite this, regenerative capacity remains limited, particularly within avascular regions.

Biomechanically, the menisci contribute to load transmission, shock absorption, joint stabilization, and lubrication, thereby protecting articular cartilage from degeneration. Loss of meniscal tissue, particularly following meniscectomy, significantly alters joint biomechanics and increases the risk of osteoarthritis. The medial meniscus also functions as a secondary stabilizer in anterior cruciate ligament-deficient knees, further underscoring the importance of meniscal preservation.

Meniscal lesions are common orthopaedic conditions with significant functional implications. They most frequently involve the posterior horn and body of the medial meniscus and may be classified as traumatic or degenerative. Degenerative meniscal tears arise from chronic overload and age-related tissue degeneration and become increasingly prevalent with advancing age [2]. Risk factors include male sex, abnormal limb alignment, discoid meniscus, elevated body mass index, occupational kneeling or squatting, and participation in high-impact activities.

Diagnosis is based on clinical assessment supported by imaging. Patients typically present with joint line tenderness, swelling, mechanical symptoms, and restricted range of motion. MRI remains the diagnostic gold standard, although degenerative imaging findings may not always correlate with clinical symptoms [3].

Management strategies depend on tear type, location, and symptom severity. Conservative treatment is generally preferred for degenerative meniscal lesions and includes physiotherapy, anti-inflammatory medication, activity modification, and injection-based therapies [4]. Surgical options, such as arthroscopic partial meniscectomy or meniscal repair, are reserved for selected cases following failed conservative management [5]. The European Society of Sports Traumatology, Knee Surgery and Arthroscopy (ESSKA) consensus emphasizes tissue preservation and highlights the association between meniscectomy and the subsequent development of osteoarthritis.

In recent years, biological therapies have emerged as potential alternatives or adjuncts to conventional treatments. Orthobiologic therapies (biologically derived substances used to support tissue repair and regeneration), such as platelet-rich plasma and hyaluronic acid, have been explored, although current evidence remains heterogeneous and sometimes conflicting [6,7]. Consequently, further high-quality studies are required to establish clear treatment guidelines. This broad characterization is consistent with recent reviews of injectable therapies for meniscal pathology and related knee conditions.

Polynucleotide (PN) gel represents a novel infiltrative treatment option with demonstrated benefits in knee osteoarthritis [8,9]. Derived from highly purified long-chain DNA polymers, PNs exhibit viscoelastic properties and form a 3D gel matrix capable of binding water molecules. Following enzymatic degradation, they yield nucleotides and nucleosides naturally present within the joint environment, suggesting potential roles in tissue protection, symptom modulation, and the stimulation of biological healing.

This scoping review aimed to evaluate the safety and effectiveness of intra- and perimeniscal PN gel injections in adults with degenerative meniscal lesions, compared with placebo or other conservative or injectable therapies. The primary objective was to map the extent, nature, and characteristics of the available clinical evidence.

A preliminary search of the literature, including the Cochrane Library, MEDLINE, PubMed, ScienceDirect, SAGE, Wiley, and Oxford Academic journals, revealed very few studies addressing this intervention. Notably, no prior systematic reviews or scoping reviews were identified, highlighting a clear gap in the synthesized evidence and the need for further investigation.

## Review

Materials and methods

Inclusion Criteria

Published human clinical studies, including randomized controlled trials, prospective and retrospective cohort studies, and case series with at least 10 participants, were eligible for inclusion. Studies involving adults (≥18 years) with degenerative, non-traumatic meniscal pathology of the knee were considered. Eligible interventions included PN-based injections (PN, polydeoxyribonucleotide (PDRN), polynucleotides highly purified technology (PN-HPT)) administered intra-articularly, perimeniscally, or directly targeting meniscal pathology. Studies were required to report at least one clinical or radiological outcome measure, including pain, functional outcomes, imaging findings, or adverse events. Only full-text articles published in English were included.

Exclusion Criteria

Animal and in vitro studies were excluded. Studies involving acute or traumatic meniscal tears, particularly in athletes or patients younger than 40 years, were not considered. Articles evaluating combined biologic treatments in which the independent effect of PNs could not be isolated were excluded. Review articles, editorials, and conference abstracts without extractable primary data were also excluded. Additionally, non-English publications without an accessible full-text translation were not included.

Search Strategy 

A comprehensive literature search was conducted on January 17, 2026 to identify studies evaluating the efficacy and safety of PN injections in adults with degenerative meniscal tears. The search was performed across databases accessible via our institution and was structured according to the Population-Concept-Context (PCC) framework. Controlled vocabulary and free-text terms related to PNs and degenerative meniscal pathology were combined using three concept lines: intervention: polynucleotide OR polydeoxyribonucleotide OR PDRN; population: adults aged >18 years; and condition: degenerative OR degeneration OR “degenerative meniscal tear” OR “meniscal degeneration,” with OR applied within each line and AND between lines.

The search strategy was developed using combinations of controlled vocabulary and free-text terms related to degenerative meniscal pathology and PN-based therapies. Both generic terms (e.g., PNs, PDRN) and potential product-related terminology were considered to maximise search sensitivity and ensure that studies published under brand-specific naming conventions were not missed.

Example PubMed search string: (“Polynucleotides”[MeSH] OR polynucleotide OR polynucleotides OR polydeoxyribonucleotide OR PDRN) AND (meniscus OR meniscal) AND (degenerative OR degeneration OR “degenerative meniscal tear”).

*Data Extraction*****

No date restrictions were applied, and the search was limited to studies published in English. Eligible study designs and population characteristics were defined according to the predefined inclusion and exclusion criteria. All records were exported into reference management software, and duplicates were removed before screening. The remaining articles were imported into Rayyan for title and abstract screening, resulting in 72 references for further assessment. Following duplicate removal, records underwent title and abstract screening and subsequent full-text assessment in accordance with the predefined eligibility criteria.

No relevant Cochrane reviews or Oxford Academic journal articles were identified, highlighting a gap in the synthesized evidence for this topic. Databases such as UpToDate, BMJ Best Practice, and other secondary or subscription-based resources were not included in the formal search because they provide summaries rather than primary studies. Furthermore, the reference list of the included study was manually screened to identify any additional eligible studies; no further relevant articles meeting the inclusion criteria were identified apart from the included study [10].

The final search was conducted across multiple electronic databases. The Cochrane Library and Oxford Academic Journals yielded no relevant results. MEDLINE (EBSCO) identified two records, while MEDLINE (Ovid) identified 10 records. PubMed returned five results. ScienceDirect yielded 25 records, SAGE Journals returned 10 records, and Wiley Online Library identified 20 records. Backward citation searching of the included study did not identify any additional eligible articles.

**Figure 1:**
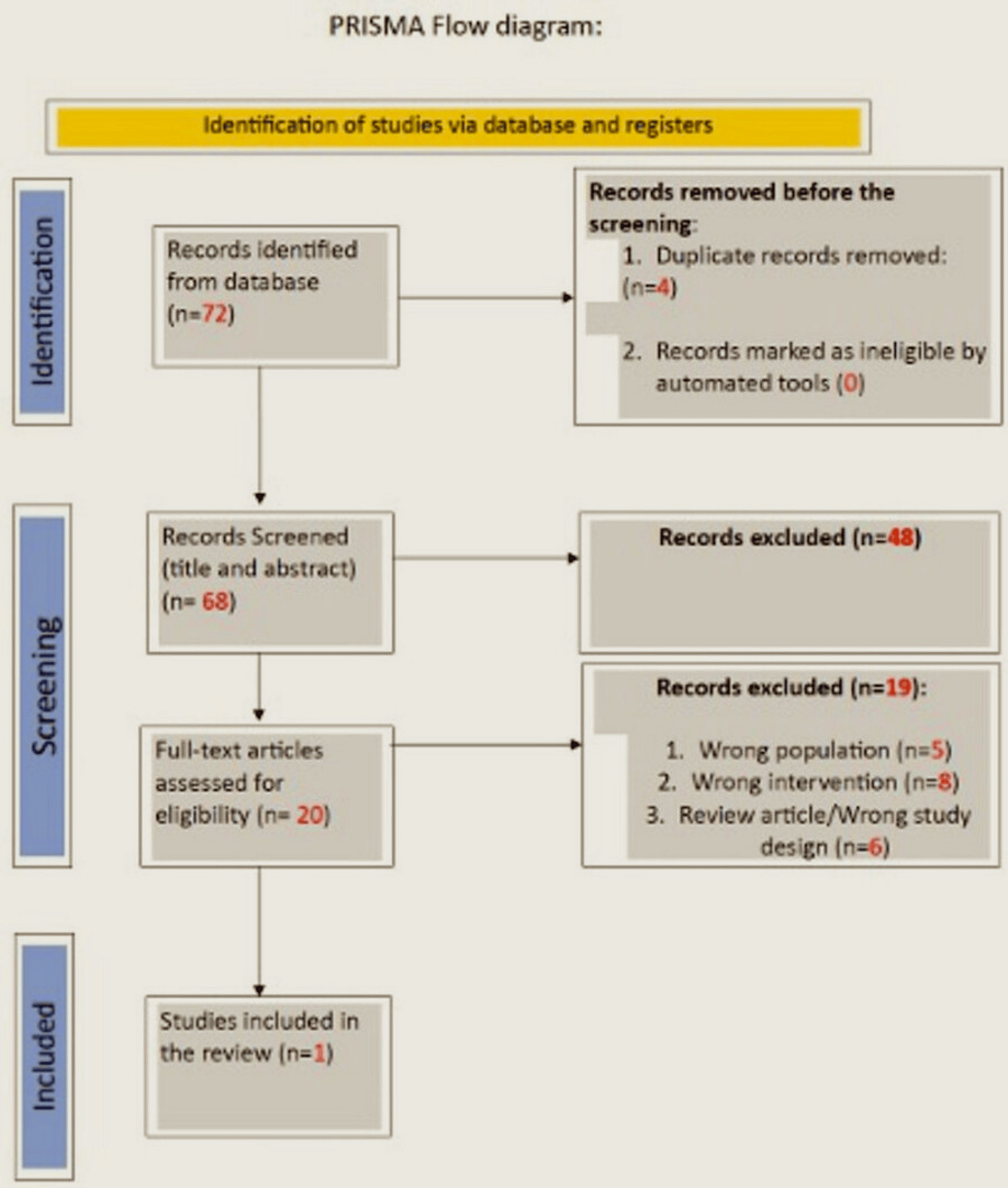
PRISMA flow diagram. Source: Reference [11] PRISMA: Preferred Reporting Items for Systematic Reviews and Meta-Analyses.

Full-text screening was performed independently by one reviewer, with uncertainties resolved through discussion with a senior clinician. Study selection was performed in accordance with PRISMA-ScR guidelines [11], and the screening process is summarised in the PRISMA flow diagram (Figure 1).

Records were identified through database searching across MEDLINE, PubMed, ScienceDirect, SAGE Journals, Wiley Online Library, the Cochrane Library, and Oxford Academic. After removal of duplicates, titles and abstracts were screened for relevance. Full-text articles were assessed for eligibility according to predefined inclusion and exclusion criteria. Studies were excluded if they did not involve degenerative meniscal pathology, did not evaluate isolated PN-based interventions, involved non-human models, or were review articles or conference abstracts without extractable data. One study met the inclusion criteria and was included in the final synthesis.

This study is a scoping review of published literature and did not involve human participants or animals. Ethical approval and informed consent were therefore not required.

The PRISMA-ScR statement [11] and the associated flow diagram template are publicly available reporting guidelines and were used in accordance with their published guidance. The VAS [12], the Cochrane Risk of Bias tool [13], the Knee Injury and Osteoarthritis Outcome Score (KOOS) [14], and the International Knee Documentation Committee (IKDC) [15] score are established, non-proprietary assessment instruments widely available for academic use. All tools employed in this study are free to use for research purposes and have been appropriately cited in the manuscript, including within relevant tables and figures where applicable.

Results

Study Selection

The database search identified 72 records across the predefined electronic databases. After the removal of duplicates, 68 studies remained and underwent title and abstract screening. Of these, 48 articles were excluded for failing to meet the inclusion criteria. Following initial screening, 20 full-text articles were assessed for eligibility. Nineteen studies were excluded at the full-text stage, primarily because they were review articles, focused on osteoarthritis rather than degenerative meniscal pathology, involved non-human models, or lacked an isolated PN-based intervention. Ultimately, one study [10] met all predefined inclusion criteria and was included in the qualitative synthesis. Given the limited number and heterogeneity of eligible studies, quantitative synthesis was not undertaken, consistent with the objectives of a scoping review.

Study Characteristics

The characteristics of the included study are summarised in Table 1 [10]. The single included study was a prospective clinical trial conducted in an adult population with symptomatic, MRI-confirmed degenerative meniscal tears. The study evaluated the clinical efficacy and safety of intra- and perimeniscal PN injections over a medium-term follow-up period. Outcome measures focused on patient-reported pain, functional scores, activity level, and adverse events [10].

**Table 1:** Characteristics of the included study. VAS: Visual Analogue Scale; KOOS: Knee Injury and Osteoarthritis Outcome Score; IKDC: International Knee Documentation Committee. Reference citations in this column indicate the source of the corresponding outcome measure.

Author (year)	Country	Study design	Sample size	Intervention	Follow-up	Outcomes reported
Anzillotti G et al. [10] (2025)	Italy	Prospective clinical trial	30 patients	Three intra- and perimeniscal polynucleotide injections	12 months	VAS pain [12], KOOS [14], IKDC [15], Tegner Activity Scale [16], adverse events

Given the limited number of eligible studies and the heterogeneity of available data, quantitative synthesis or meta-analysis was not undertaken, and the findings were summarized descriptively.

Outcomes

Pain was assessed using the VAS [12]. The included prospective study enrolled 30 patients with MRI-confirmed degenerative meniscal tears, of whom 24 completed the 12-month follow-up. Clinically meaningful improvements were observed across multiple outcome measures. Mean KOOS scores [14] improved from 61.99 at baseline to 84.32 at 12 months, while VAS pain scores [12] decreased from 62.68 to 20.63 over the same period. Functional outcomes also improved, with IKDC scores [15] increasing from 46.39 to 72.46 and Tegner activity scores rising from 2.32 to 4.50. No serious adverse events related to the injections were reported. Minor, self-limiting local reactions at the injection site were described, and no patients required surgical intervention during follow-up.

Risk of Bias 

The included study was a non-randomized prospective clinical investigation without a control group, placing it at moderate risk of bias. The primary sources of bias included the absence of randomization and blinding, as well as reliance on patient-reported outcome measures. However, the use of validated scoring systems and acceptable completeness of follow-up partially mitigated these limitations. As the included study was a non-randomized prospective clinical study without a comparator group, the formal application of the Cochrane Risk of Bias tool designed for randomized trials was limited. Therefore, the risk of bias was assessed qualitatively by considering potential sources of bias related to study design, patient selection, outcome measurement, and reporting [13].

Discussion

The principal finding of this scoping review is that the evidence evaluating the efficacy and safety of PN injections for degenerative meniscal tears is very limited. Only one prospective clinical study met the predefined inclusion criteria, reflecting the emerging nature of this therapeutic approach. Despite the scarcity of data, the included study reported clinically meaningful improvements in pain, knee function, and activity level following intra- and perimeniscal PN injections, with no serious treatment-related adverse events observed.

Degenerative meniscal tears represent a common source of knee pain and functional limitation, particularly in middle-aged and older adults. Conservative management remains the cornerstone of treatment, with growing interest in injectable therapies aimed at symptom control and tissue preservation. Hyaluronic acid and platelet-rich plasma are among the most widely investigated orthobiologics in this context; however, results across studies remain heterogeneous, and clear consensus regarding their efficacy is lacking. PN-based injectables have recently gained attention due to their viscoelastic properties, water-binding capacity, and potential biological effects on the joint microenvironment. While these properties have been explored primarily in knee osteoarthritis, their application to meniscal pathology remains largely unexplored.

The findings of the included study suggest that PN injections may offer symptomatic benefit in patients with degenerative meniscal lesions, potentially through a combination of mechanical cushioning, modulation of the intra-articular environment, and stimulation of reparative processes. Improvements in validated patient-reported outcome measures, including VAS [12], KOOS [14], IKDC [15], and Tegner scores [16], indicate potential clinical relevance, particularly for patients seeking non-surgical treatment options. Importantly, the reported safety profile was favourable, with no major adverse events or progression to surgical intervention during follow-up, supporting the tolerability of this approach.

However, the interpretation of these findings must be tempered by the methodological limitations of the available evidence. The included study was non-randomized and lacked a control group, introducing an inherent risk of bias and limiting causal inference. Reliance on patient-reported outcomes, although clinically meaningful, may be influenced by placebo effects and expectation bias. In addition, the small sample size and single-study design preclude meaningful comparison with other injectable therapies and prevent quantitative synthesis of results. As such, the current evidence is insufficient to draw definitive conclusions regarding the superiority or comparative effectiveness of PN injections in degenerative meniscal pathology.

This review highlights a substantial gap in the literature and underscores the need for further high-quality research. Future studies should prioritise randomized controlled trial designs with appropriate comparator groups, standardized injection protocols, and clearly defined outcome measures. Longer follow-up periods and the incorporation of imaging-based assessments may help clarify the potential structural or disease-modifying effects of PN therapy. Comparative studies against established injectables, such as hyaluronic acid or platelet-rich plasma, would also be valuable in defining the role of PNs within the current treatment algorithm.

A key limitation of this review is that only one study met the predefined inclusion criteria. While this reflects the current scarcity of published clinical research investigating PN-based injections for degenerative meniscal pathology, it limits the ability to draw firm conclusions or perform comparative synthesis across multiple studies. Consequently, the findings should be interpreted cautiously. The limited evidence base highlights the need for further well-designed prospective and randomized studies to better evaluate the clinical effectiveness and safety of PN-based therapies in this patient population.

The search was conducted up to January 2026; therefore, studies published after this date were not included in the analysis.

In summary, while preliminary evidence suggests that PN injections may be a safe and potentially effective conservative treatment for degenerative meniscal tears, the current body of evidence remains limited. Well-designed clinical trials are required before this intervention can be routinely recommended in clinical practice.

## Conclusions

PN injections represent a promising conservative treatment option for adults with degenerative meniscal tears; however, the current evidence base remains limited. This scoping review identified only one eligible prospective clinical study, which reported improvements in pain, knee function, and activity level, alongside a favourable safety profile. While these preliminary findings suggest potential clinical benefit, the absence of randomized controlled trials and comparative data precludes definitive conclusions regarding efficacy and is insufficient to support routine clinical adoption. Further well-designed studies with appropriate control groups, standardized protocols, and longer follow-up are required to clarify the role of PN injections in the management of degenerative meniscal pathology.
